# A chest CT-based nomogram for predicting survival in acute myeloid leukemia

**DOI:** 10.1186/s12885-024-12188-8

**Published:** 2024-04-12

**Authors:** Xiaoping Yi, Huien Zhan, Jun Lyu, Juan Du, Min Dai, Min Zhao, Yu Zhang, Cheng Zhou, Xin Xu, Yi Fan, Lin Li, Baoxia Dong, Xinya Jiang, Zeyu Xiao, Jihao Zhou, Minyi Zhao, Jian Zhang, Yan Fu, Tingting Chen, Yang Xu, Jie Tian, Qifa Liu, Hui Zeng

**Affiliations:** 1 Department of Radiology, Xiangya Hospital, Central South University, Changsha, China; 2 Department of Hematology, The First Affiliated Hospital of Jinan University, Guangzhou, China; 3 Department of Clinical Research, The First Affiliated Hospital of Jinan University, Guangzhou, China; 4 Department of Hematology, Nanfang Hospital, Southern Medical University, Guangzhou, China; 5 Department of Nuclear Medicine, The Third Xiangya Hospital, Central South University, Changsha, China; 6 Department of Hematology, Xiangya Hospital, Central South University, Changsha, China; 7 Department of Geriatrics, Guangzhou First People’s Hospital, School of Medicine, South China University of Technology, Guangzhou, China; 8 Department of Hematology, The First Affiliated Hospital of Soochow University, Suzhou, China; 9 Department of Hematology, Hunan Provincial People’ Hospital, The First Affiliated Hospital of Hunan Normal University, Changsha, China; 10 Department of Hematology, Shanghai General Hospital, Shanghai Jiaotong University School of Medicine, Shanghai,, China; 11 The Guangzhou Key Laboratory of Molecular and Functional Imaging for Clinical Translation, The First Affiliated Hospital of Jinan University, Guangzhou, China; 12 Department of Hematology, Shenzhen People’s Hospital, Second Clinical Medical College of Jinan University, First Affiliated Hospital of Southern University of Science and Technology, Shenzhen, China; 13 Department of Hematology, The Seventh Affiliated Hospital, Sun Yat-sen University, Shenzhen, China; 14 Department of Hematology, The Third Xiangya hospital, Central South University, Changsha, China; 15 CAS Key Laboratory of Molecular Imaging, Institute of Automation, Chinese Academy of Sciences, Beijing, China; 16 National Engineering Research Center of Personalized Diagnostic and Therapeutic Technology, Xiangya Hospital, Changsha, China; 17 National Clinical Research Center for Geriatric Disorders (Xiangya Hospital), Central South University, Changsha, China; 18 Hunan Key Laboratory of Skin Cancer and Psoriasis, Xiangya Hospital, Central South University, Changsha, China; 19 Hunan Engineering Research Center of Skin Health and Disease, Xiangya Hospital, Central South University, Changsha, China; 20 Department of Dermatology, Xiangya Hospital, Central South University, Changsha, China

**Keywords:** Chest computed tomography, CT-derived features, Acute myeloid leukemia, Nomogram, Prognosis

## Abstract

**Background:**

The identification of survival predictors is crucial for early intervention to improve outcome in acute myeloid leukemia (AML). This study aim to identify chest computed tomography (CT)-derived features to predict prognosis for acute myeloid leukemia (AML).

**Methods:**

952 patients with pathologically-confirmed AML were retrospectively enrolled between 2010 and 2020. CT-derived features (including body composition and subcutaneous fat features), were obtained from the initial chest CT images and were used to build models to predict the prognosis. A CT-derived MSF nomogram was constructed using multivariate Cox regression incorporating CT-based features. The performance of the prediction models was assessed with discrimination, calibration, decision curves and improvements.

**Results:**

Three CT-derived features, including myosarcopenia, spleen_CTV, and SF_CTV (MSF) were identified as the independent predictors for prognosis in AML (*P* < 0.01). A CT-MSF nomogram showed a performance with AUCs of 0.717, 0.794, 0.796 and 0.792 for predicting the 1-, 2-, 3-, and 5-year overall survival (OS) probabilities in the validation cohort, which were significantly higher than the ELN risk model. Moreover, a new MSN stratification system (MSF nomogram plus ELN risk model) could stratify patients into new high, intermediate and low risk group. Patients with high MSN risk may benefit from intensive treatment (*P* = 0.0011).

**Conclusions:**

In summary, the chest CT-MSF nomogram, integrating myosarcopenia, spleen_CTV, and SF_CTV features, could be used to predict prognosis of AML.

**Supplementary Information:**

The online version contains supplementary material available at 10.1186/s12885-024-12188-8.

## Introduction

Acute myeloid leukemia (AML) is the common hematological malignancy comprising approximately 15% of leukemia cases. AML is driven by a series of genetic and epigenetic events, with induction chemotherapy and hematopoietic stem cell transplantation remaining the standard treatments [1, 2]. Despite a relatively high response rate, the prognosis of AML patients varies. The risk factors for AML with poor prognosis include clinical demographics, blood cell counts, serum oncological indicators, protein expression, and gene profiles [3, 4]. However, there is still an urgent need for new noninvasive, objective and time-efficient methods to identify those with poor prognosis and aid choice of therapy.

Chest computed tomography (CT) is the most widely used imaging method for routinely assessing lung conditions in all malignant diseases, including AML. However, it is challenging to predict prognostic status based on the traditional radiological evaluation of CT images in AML. CT based body composition evaluation, such as of myosarcopenia, refers to the computational extraction and analysis of imaging features from clinically acquired radiological images. Sarcopenia (loss of lean muscle mass) is an important reason for longer hospital stays and premature mortality in patients with nonmalignant disease [5]. Recently, the preliminary prognostic impact of sarcopenia has also been highlighted in AML [6–8] and acute lymphoblastic leukemia patients [9], demonstrating the potential for imaging-based prognostic prediction in leukemia. Notably, these studies were all conducted based on abdominal CT imaging. Unfortunately, AML patients do not routinely undergo abdominal CT scans unless they have coexisting abdominal diseases. Instead, a pretreatment chest CT is performed for AML patients prior to hospitalization in many institutions. Nevertheless, whether it is feasible to quantify CT-derived features based on pretreatment chest CT for the prognosis of AML patients is still unknown.

## Methods

### Patients

**Fig. 1:**
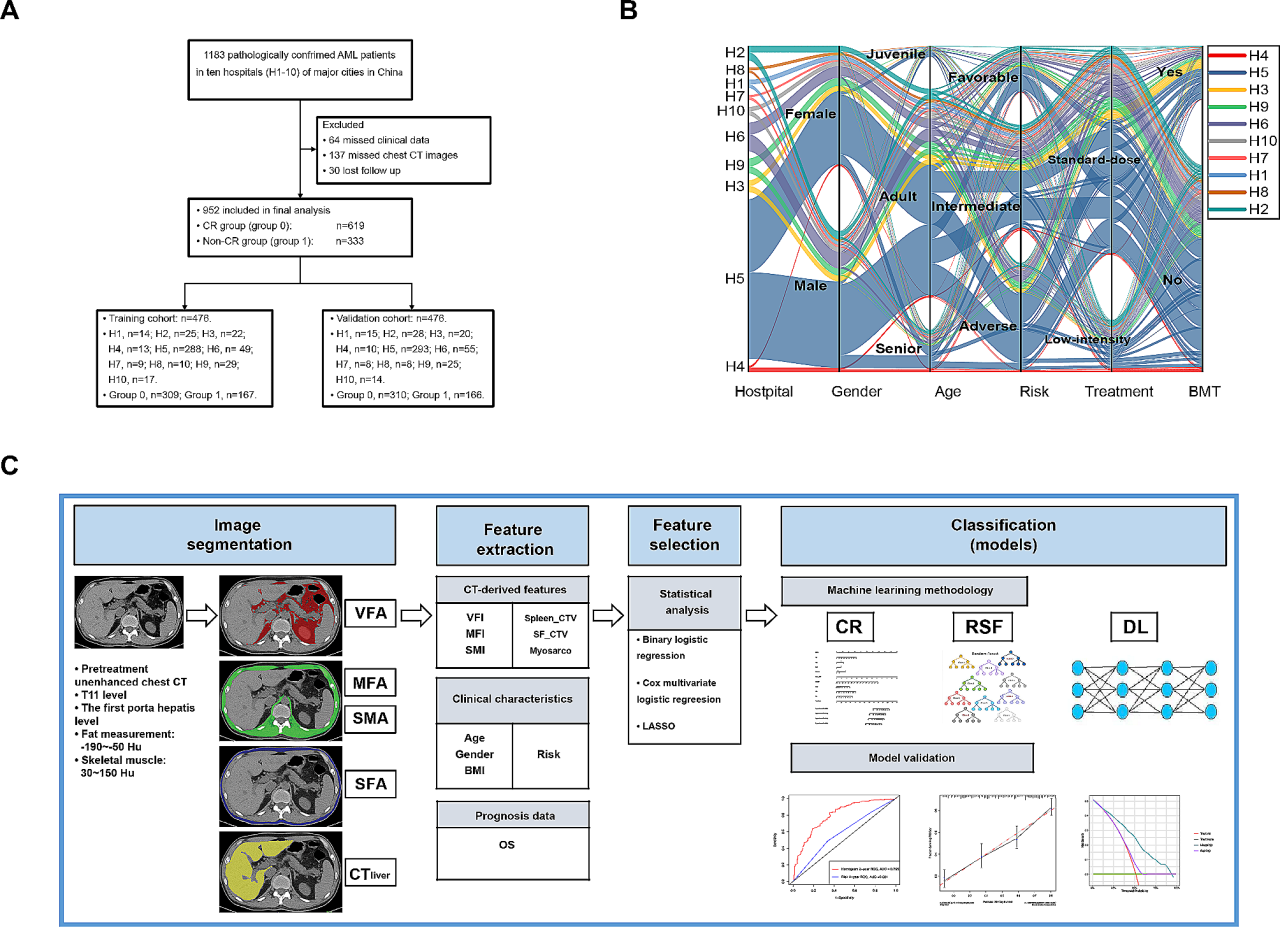
Enrollment, clinicopathological characteristics and body composition assessment of AML patients. (**A**) Flow-chart demonstrating the exclusion criteria and the patient recruitment process with the reason for exclusion. (**B**) Sankey diagram showing the scmap cluster projection of the key characteristics of 952 patients used in this study, including hospital, gender, age composition, risk, treatment response and BMT status. (**C**) Workflow of the body composition feature- and image-based analysis in this study. VFA, visceral fat area; MFA, muscle fat area; SMA, skeletal muscle area; SFA, subcutaneous fat area; Liver_CTV, CT value of liver parenchyma; VFI, visceral fat index; MFI, muscle fat index; SMI, skeletal muscle index; Spleen_CTV, spleen CT value; SF_CTV, subcutaneous fat CT value; myosarco, myosarcopenia. Risk, ELN risk; BMT, bone marrow transplantation; OS, overall survival; CR, Cox proportional-hazards regression; RSF, random survival forest; DL, deep learning.

Through an evaluation of our institutional medical database from January 2010 to February 2020, data were retrospectively collected for *de novo* AML diagnosed in accordance with WHO criteria in ten participating hospitals. Details of the patient recruitment process and exclusion criteria are shown in Fig. 1. All 952 patients were randomly allocated into a training cohort (476 patients) and a validation cohort (476 patients). The general information of patients in these two cohorts is presented in Table 1.

**Table 1:** Clinical and body composition status data of 952 leukemia cases

Characteristics	All samples (*n* = 952)	Training (*n* = 476)	Validation (*n* = 476)	P Value
Sex (n, %)				0.696
Female	438 (46.01)	222 (46.6)	216 (45.4)	
Male	514 (53.99)	254 (53.4)	260 (54.6)	
Subtype (n, %)				0.547
M0	12 (1.26)	7 (1.50)	5 (1.10)	
M1	56 (5.88)	23 (4.80)	33 (6.90)	
M2	465 (48.84)	234 (49.20)	231 (48.50)	
M3	36 (3.78)	21 (4.40)	15 (3.20)	
M4	217 (22.79)	114 (23.90)	103 (21.60)	
M5	161 (16.91)	74 (15.50)	87 (18.30)	
M6	5 (0.53)	3 (0.60)	2 (0.40)	
Treatment (n, %)				0.105
Standard-dose	762 (80.04)	371 (77.90)	391 (82.10)	
Low-intensity	190 (19.96)	105 (22.10)	85 (17.90)	
ELN 2022 Risk (n, %)				0.743
Adverse	398 (41.81)	202 (42.44)	196 (41.18)	
Intermediate	385 (40.44)	194 (40.76)	191 (40.12)	
Favorable	169 (17.75)	80 (16.8)	89 (18.70)	
BMT				0.81
Non- Transplantation	755 (79.31)	379 (79.60)	376 (79.00)	
Transplantation	197 (20.69)	97 (20.40)	100 (21.00)	
Age (median, range)	45 (32, 55)	46 (33, 56)	44 (32, 55)	0.649
BMI	22 (19.98, 24.23)	22.03 (20.1, 24.30)	21.97 (19.82, 24.11)	0.306
SP (mmHg)	115.50 (107.00, 125.00)	115.0 (107.0, 124.75)	116.0 (107.0, 125.00)	0.812
DP (mmHg)	72 (65, 79)	71 (65, 78)	72 (66, 79)	0.587
WBC (×109/L)	16.39 (5.51, 46.29)	16.42 (4.93, 45.29)	16.32 (5.88, 48.68)	0.646
HGB(g/L)	74.00 (62.00, 92.00)	73.50 (62.0, 91.75)	74.00 (61.00,92.00)	0.904
PLT (×109/L)	43.00 (22.00, 80.00)	43.50 (21.00, 86.75)	42.00(23.00, 76.75)	0.924
Neu (×109/L)	2.10 (0.57, 7.79)	2.00 (0.56, 7.60)	2.1 ( 0.55, 8.72)	0.95
Lym (×109/L)	3.63 (1.40, 10.16)	3.53 (1.37, 10.48)	3.79 (1.40, 9.36)	0.928
Mono (×109/L)	4.11 (0.5, 19.4)	3.83 (0.50, 19.40)	4.34 (0.51, 19.55)	0.871
NLR (%)	0.64 (0.19, 1.76)	0.61 (0.19, 1.76)	0.65 (0.18, 1.80)	0.977
LMR (%)	0.91 (0.34, 4.76)	0.89 (0.34, 4.80)	0.93 (0.34, 4.55)	0.922
PLR (%)	10.93 (3.68, 38.97)	10.96 (3.63, 41.20)	10.85 (3.72, 35.79)	0.749
RDW (fl.)	60.40 (50.70, 88.20)	60.70 (50.80, 88.28)	59.95 (50.63, 88.15)	0.548
MPV (fl.)	10.30 (9.20, 11.90)	10.20 (9.04, 11.80)	10.50 (9.30, 12.00)	0.133
Albumin (g/L)	37.20 (33.30, 41.00)	36.85 (33.05, 40.60)	37.60 (3.40, 41.68)	0.121
Globulin (g/L)	28.50 (24.60, 33.20)	28.40 (24.65, 32.88)	28.50 (24.45, 33.28)	0.59
AGR	1.31 (1.07, 1.57)	1.30 (1.07, 1.56)	1.30 (1.07, 1.58)	0.521
HDL (mmol/L)	0.84 (0.71, 0.97)	0.85 (0.72, 0.98)	0.83 (0.68, 0.97)	0.074
LDL (mmol/L)	1.92 (1.50, 2.50)	1.93 (1.52, 2.52)	1.91 (1.48, 2.47)	0.527
TG (mmol/L)	1.46 (1.07, 2.06)	1.46 (1.08, 2.03)	1.43 (1.02, 2.12)	0.934
TC (mmol/L)	3.32 (2.75, 4.15)	3.34 (2.77, 4.14)	3.31 (2.70, 4.18)	0.882
BUN(mmol/L)	4.34 (3.30, 5.59)	4.38 (3.38, 5.60)	4.30 (3.25, 5.51)	0.277
Scr (umol/L)	71.00 (59.00,86.00)	73.00 (60.00, 87.00)	70.00 (58.00, 85.00)	0.085
BG	5.67 (5.10, 6.76)	5.67 (5.10, 6.75)	5.67 (5.10, 6.77)	0.937
LDH(U/L)	511.00 (296.80, 862.00)	519.10 (307.00, 872.00)	497.50 (285.53, 846.00)	0.395
BM(blast)	0.61 (0.39, 0.79)	0.61 (0.41, 0.77)	0.61 (0.37, 0.81)	0.989

*Note*—Unless otherwise indicated, data are numbers of patients, and data in parentheses are

percentages. # age is presented as median (minimum ∼ maximum)

### Acquisition and retrieval procedure of CT images and radiological and body composition feature extraction

CT image acquisition, the image retrieval procedure, the algorithms for radiological and body composition feature extraction, and intra-observer (reader 1 twice) and inter-observer (reader 1 vs. reader 2) reproducibility evaluation were performed as previously described [10]. Briefly, CT-derived features, including skeletal muscle index (SMI), skeletal muscle radiation attenuation (SM-RA), liver CT value (liver_CTV) (Housfield units, HU), visceral or subcutaneous fat index (VFI or SFI), spleen CT value (spleen_CTV), and subcutaneous fat CT value (SF_CTV) evaluated by reader 1 were obtained from the initial chest CT images at the level of the fourth thoracic vertebra (T4) and were used to build models to predict the prognosis of AML.

### Development of an individualized prediction model

The multivariable Cox regression method, which is suitable for the regression of medical data, was performed based on the candidate clinical and radiological predictors. Briefly, LASSO regression and clinical experience was used to screen for correlation factors [11]. Then, a prognostic model for predicting the 1-, 2-, 3-, and 5-year OS probabilities was developed by Cox regression. A predictive model was also constructed by using an ensemble machine learning method or deep learning algorithm. Details of machine learning and deep learning methods can be found in the supplementary methods.

### Performance, validation and clinical use of the nomogram

The calibration of the nomogram was evaluated by calibration curves, and the diagnostic efficiency was quantified with Harrell’s C-index.

The performance of the nomogram was tested in the validation cohort with a series of indicators [12]. The concordance index (C-index) and the area under the time-dependent receiver operating characteristic (ROC) curve (AUC) were used to evaluate the differentiation ability of the new model. The performance of the new nomogram was further supplemented with two more indicators (net reclassification improvement [NRI] and integrated discrimination improvement [IDI]) to increase the accuracy and comprehensiveness of the comparisons. The consistency between the survival probabilities predicted using the nomogram and the actual result was evaluated by drawing calibration plots. Finally, decision-curve analysis (DCA) was performed to evaluate the clinical validity of the model. The total scores on the nomogram were divided into high-, and low- risk groups by X-Tile software. The MSN risk groups were identified by combining the CT-MSF nomogram risk and the ELN risk.

### Statistical analysis

All of the statistical analysis were performed using IBM SPSS Statistics software (version 27.0, SPSS, Chicago, IL, USA), R software (version 4.0.3; http://www.Rproject.org) and X-tile software (version 3.6.1; http://tissuearray.org/). A bilateral probability value of *p* < 0.05 was considered indicative of statistical significance. *, *P* < 0.05. **, *P* < 0.01. ***, *P* < 0.001.

### Data availability

The data and code of this study are available from the corresponding author upon request.

## Results

### Enrollment, characteristics and CT-derived features of the AML patients

A total of 952 AML patients at ten different hospitals (H1-10), were retrospectively included in this study. These patients were randomly assigned into the training cohort and validation cohort (Fig. 1A). Characteristics of these patients were presented in Fig. 1B; Table 1. No significant difference was observed in any parameters. Image pre-processing and analysis, prediction model construction process were performed as shown in Fig. 1C.

### Establishment and evaluation of the CT-MSF model

**Fig. 2:**
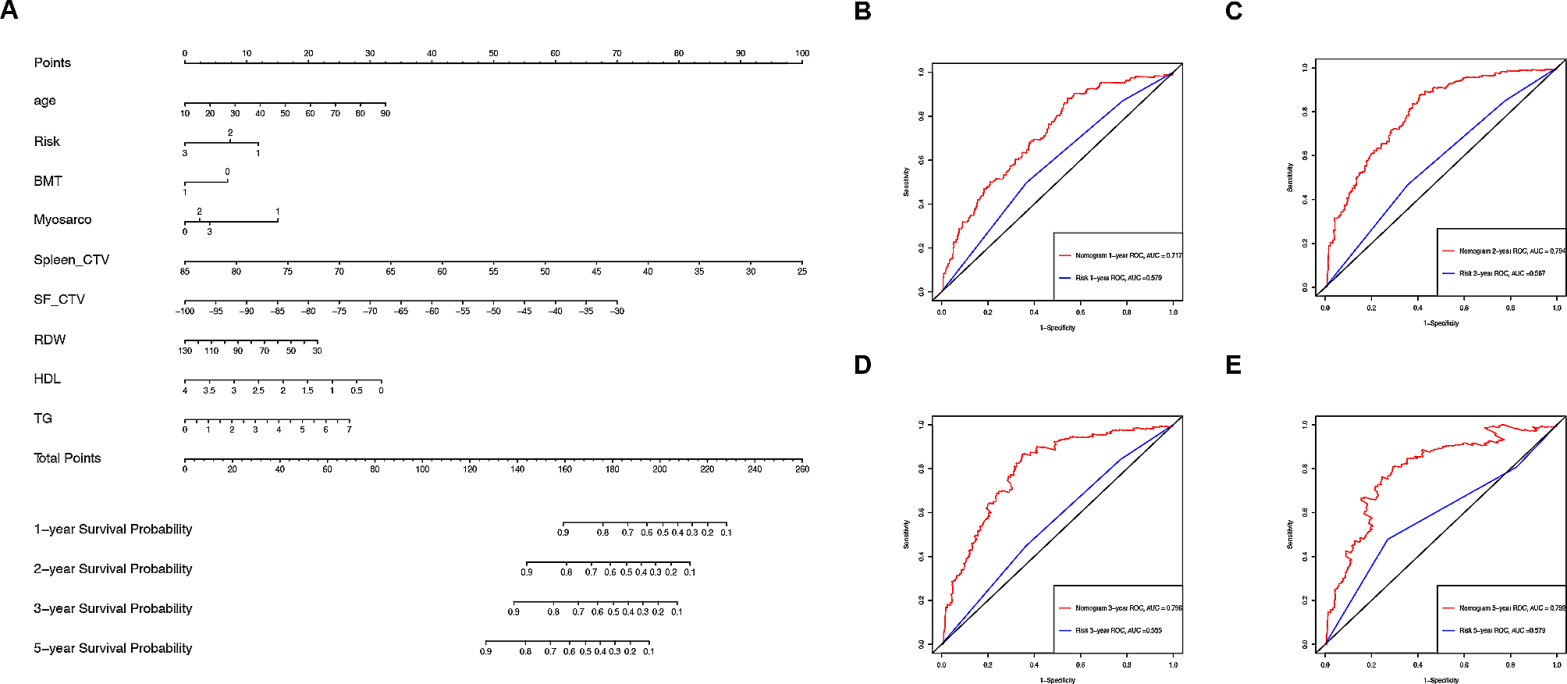
Development and validation of CT-MSF Prediction Model. (**A**) CT-based MSF nomogram for the prognosis of AML. The CT-based MSF model was developed in the training cohort, incorporating the CT-derived parameters including myosarco, Spleen_CTV, and SF-CTV. Myosarco, myosarcopenia. Spleen_CTV, spleen CT value. SF-CTV, subcutaneous fat CT value. RDW, red blood cell distribution width. HDL, high-density lipoproteins. TG, triacylglyceride. Risk1, ELN high risk. Risk2, ELN intermediate risk. Risk3, ELN low risk. BMT 0, no BMT. BMT 1,with BMT. Myosarco 0, no sarcopenia. Myosarco 1, myosarcopenia. Myosarco 2, myosteatosis. Myosarco 3, sarcopenia. (**B**-**E**) Receiver operating characteristic (ROC) curves for validation of the CT-MSF model (red) and ELN risk model (blue) at 1 year (**B**), 2 years (**C**), 3 years (**D**) and 5 years(**E**). The area under curves (AUCs) for both models in the validation cohort of 476 patients are shown. Nomogram, the CT-MSF model. Risk, the ELN risk model.

To identify prognostic factors for AML, we performed LASSO regression analysis with clinicopathological characteristics and CT-derived features as variables. The LASSO regression analysis indicated that myosarcopenia, bone marrow transplantation (BMT), risk, BMT, spleen CT value (spleen_CTV), subcutaneous fat CT value (SF_CTV), age, red blood cell distribution width (RDW), high-density lipoprotein (HDL) and triglyceride (TG) were independent predictors for overall survival (OS) (Fig. 2A, supplementary Table 1). By multivariate Cox regression analysis, we established a new model integrating myosarcopenia, spleen_CTV and SF_CTV (CT-MSF). The CT-MSF nomogram showed a performance with a C index of 0.735 and 0.690 for the training and validation cohort, which were significantly higher than the C index of 0.557 and 0.557 for the ELN risk model (*P* < 0.05). Moreover, this predictive model achieved AUCs of 0.717, 0.794, 0.796 and 0.792 for predicting the 1-, 2-, 3- and 5-year OS probabilities in the validation cohort, respectively. When compared to the traditional ELN risk model (blue), the prediction performance of CT-MSF model (red) had significantly better predictive performance (Fig. 2B-E). Interestingly, the CT-MSF nomogram (cyan) added more benefit than the ELN risk model (purple) (Figure S1). The calibration curves of the nomogram for the probability of OS showed good agreement between prediction and observation in the validation cohort (Figure S2). The net reclassification improvement (NRI) and integrated discrimination improvement (IDI) also indicated that the CT-MSF model achieved satisfactory efficiency (supplementary Table 2). Machine learning and deep learning models were also constructed but did not show better performance than the traditional Cox method included above (data not shown). Collectively, we have identified independent predictors and developed a CT-MSF nomogram to predict prognosis of AML with high accuracy.

We next assessed whether the CT-MSF model could be used to predict overall survival by applying the model to the whole dataset. Based on the scores generated by our model, all cases were classified into the CT-MSF high-risk and CT-MSF low-risk groups (CT-MSF risk), by using the X-title method. As expected, patients within the high-risk group had shorter survival than that of the low-risk patients (*p* < 0.0001, log-rank test) (Figure S3A, supplementary Table 3). We also observed that the CT-MSF high-risk patients demonstrated higher cumulative hazard than the low-risk group (Figure S3B).

### Stratification of AML patients by a combination of CT-MSF and ELN model

**Fig. 3:**
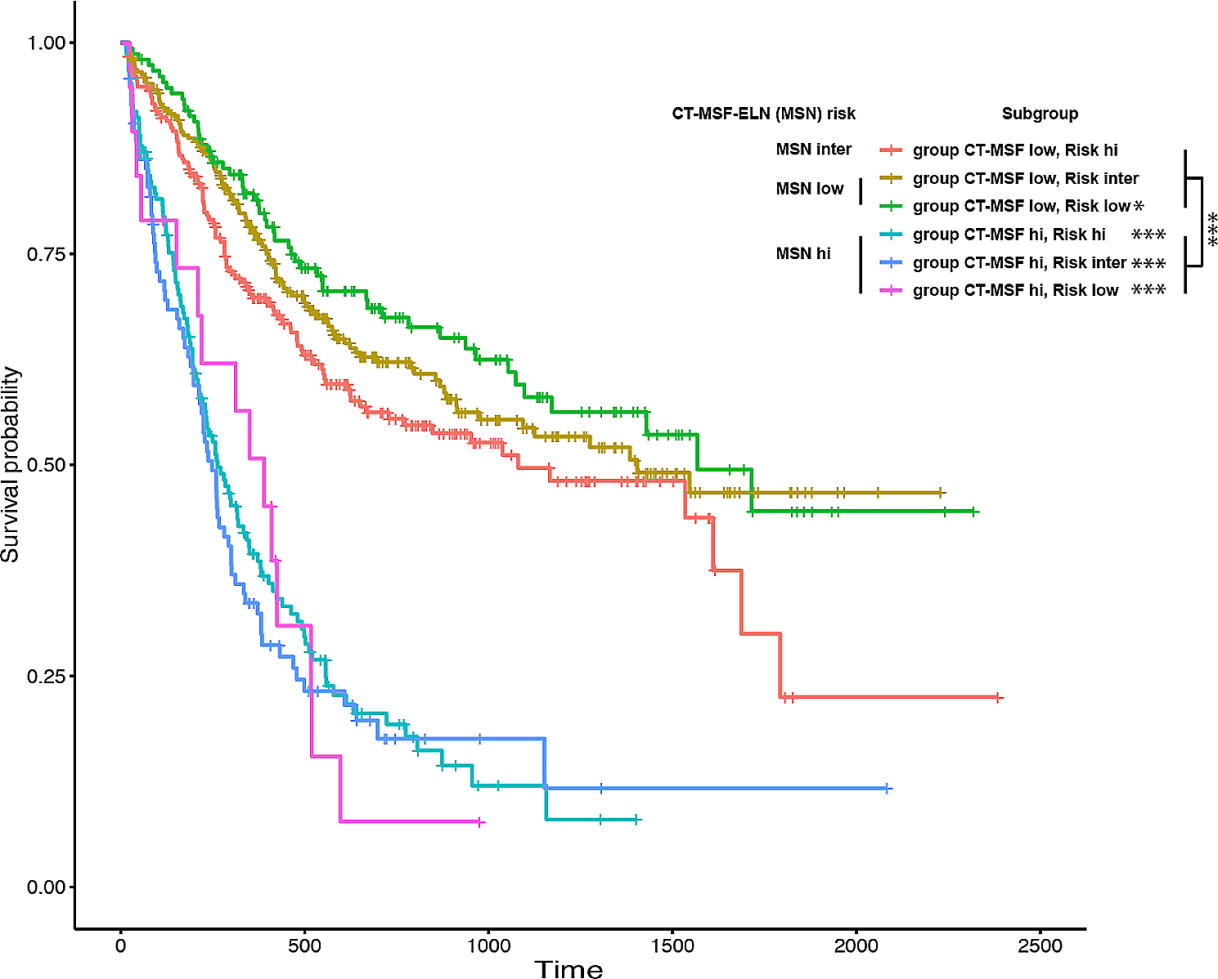
Overall survival of 952 patients stratified with the combination CT-MSF model and ELN risk model. Low, low risk. Inter, intermediate risk. Hi, high risk. *, *P* < 0.05. ***, *P* < 0.001

Next, we plotted the overall survival of this cohort with the combination of risk subgroups determined by the CT-MSF model and the ELN risk model to stratify these 952 patients. Interestingly, the survival curves fell mainly into 3 new groups. The CT-MSF low-risk and ELN low- and intermediate-risk groups formed a new MSN low-risk group with better survival. The CT-MSF low-risk/ELN high-risk group composed the new MSN intermediate-risk group, which had a medium survival time. Notably, all the CT-MSF high-risk group patients fell into the new MSF-ELN (MSN) high-risk group and had worse survival, regardless of their ELN risk (Fig. 3). Taken together, these results indicate that our CT-MSF model could further stratify patients when combined with the ELN risk model.

To clarify whether the effect of intensive and low-intensity treatment could be predicted by this new MSN risk score, we conducted prognostic analysis of patients who underwent different treatment choice. The results showed that the overall survival of patients with low and intermediate MSN risk was not affected by the choice of chemotherapy (Figure S4A and B). In contrast, among the MSN high-risk patients, the survival time of patients receiving intensive treatment was significantly longer than the low-intensity group (*P* <0.05, Figure S4C). Our data indicated that the new MSN stratification system combining the CT-MSF model and ELN risk model aid in choosing the therapy.

## Discussion

Our findings provide preliminary data to support the inclusion of CT-derived body composition parameters, sarcopenia for example, as biomarkers of prognosis in AML. Recent studies have reported that sarcopenia had adverse implications, including the increased severity of the disease, longer hospital stays, more complications, earlier postoperative recurrence, and poor prognosis [6–8]. Our findings were generally in line with these prior studies. Moreover, our model included Spleen_CTV and SF_CTV in addition to sarcopenic features. Consistently, these parameters are known to be related to prognosis in AML. A lower Spleen_CTV reflects decreased spleen density due to multiple complex reasons, including systemic inflammation, abnormal fat deposition, tumor cell infiltration, and spleen tissue cell damage [13]. The increased SF_CTV may be caused by various reasons, with systemic inflammation as the most common contributor. Therefore, myosarcopenia, spleen-CTV and SF-CTV can be used to predict the overall treatment response, representing multiscale biological characteristics associated with metabolic status, gene status, and various pathological conditions, which could potentially predict prognosis in patients with AML.

Our model could predict prognosis better than ELN risk model alone, as evidenced by our AUC values, C-index, NRI, IDI as well as DCA in the validation cohorts. We speculate that the improved model performance in our study may be due to our research strategy. First, we enrolled 952 patients from ten hospitals and pretreatment chest CT images is commonly available in all hospitals in China. Moreover, the CT images used in our cohort were obtained from different scanners with the same non-enhanced scanning protocol, which largely considered imaging variability. Finally, in addition to Cox regression, we also tried the other algorithms including ensemble machine learning classification method and deep learning to select the best method. Our study showed that the Cox model with optimization algorithm has better expressiveness and is not inferior to all the rest models in performance.

In addition to CT-derived features, our MSF nomogram also includes clinical characteristics that yet to be included in prognostic prediction models of AML, including RDW, HDL and TG. RDW is traditionally considered as a marker of the differential diagnosis of anemia, and it has been reported as a prognostic factor in AML [14]. Increased RDW is related to oxidative stress, poor nutritional status and older age and may also suggest a proinflammatory state [15]. HDL and TG are both indicators of lipid metabolism and HDL levels have been implied to be associated with the pathogenesis of AML [16]. Dysregulated lipid metabolism has been reported to be involved in the pathogenesis of AML, and several key enzymes involved in lipid synthesis have been studied and explored as the targets to treat cancers, HMGCR for example [16–18]. Consistently, these three traits serve as independent factors and important contributing factors in our nomogram.

However, there were limitations to this study. First, though we adopted a multicenter design in the present study, potential selection bias was unavoidable. In addition, the retrospective nature limits our exploration of the imaging and pathological correlation. Moreover, our sample size was still modest. In the future, a larger independent, prospective, multicenter study is needed for validation.

## Conclusions

In summary, we presented an MSF nomogram utilizing chest CT images to predict prognosis in patients with AML. The combination of the MSF nomogram and ELN risk yielded an MSN stratification system that could stratify AML patients. Moreover, this MSN stratification system could be used to assist in the choice of therapy to achieve better outcomes. The information from the current study could be used to assist clinicians in selecting optimal therapies for personalized treatment of AML.

### Electronic supplementary material

Below is the link to the electronic supplementary material.

Supplementary Material 1

## Data Availability

The data and code of this study are available from the corresponding author upon request.

## Abbreviations

AML: acute myeloid leukemia
AUC: area under curve
MSF: myosarcopenia, spleen_CTV, and SF_CTV
Spleen_CTV: spleen CT value
SF_CTV: subcutaneous fat CT value
OS: overall survival
DCA: decision curve analysis
NRI: net reclassification improvement
IDI: integrated discrimination improvement
