# Correction: A chest CT-based nomogram for predicting survival in acute myeloid leukemia

**DOI:** 10.1186/s12885-024-12323-5

**Published:** 2024-05-06

**Authors:** Xiaoping Yi, Huien Zhan, Jun Lyu, Juan Du, Min Dai, Min Zhao, Yu Zhang, Cheng Zhou, Xin Xu, Yi Fan, Lin Li, Baoxia Dong, Xinya Jiang, Zeyu Xiao, Jihao Zhou, Minyi Zhao, Jian Zhang, Yan Fu, Tingting Chen, Yang Xu, Jie Tian, Qifa Liu, Hui Zeng

**Affiliations:** 1 Department of Radiology, Xiangya Hospital, Central South University, Changsha, China; 2 Department of Hematology, The First Affiliated Hospital of Jinan University, Guangzhou, China; 3 Department of Clinical Research, The First Affiliated Hospital of Jinan University, Guangzhou, China; 4 Department of Hematology, Nanfang Hospital, Southern Medical University, Guangzhou, China; 5 Department of Nuclear Medicine, The Third Xiangya Hospital, Central South University, Changsha, China; 6 Department of Hematology, Xiangya Hospital, Central South University, Changsha, China; 7 Department of Geriatrics, Guangzhou First People’s Hospital, School of Medicine, South China University of Technology, Guangzhou, China; 8 Department of Hematology, The First Affiliated Hospital of Soochow University, Suzhou, China; 9 Department of Hematology, Hunan Provincial People’ Hospital, The First Affiliated Hospital of Hunan Normal University, Changsha, China; 10 Department of Hematology, Shanghai General Hospital, Shanghai Jiaotong University School of Medicine, Shanghai, China; 11 The Guangzhou Key Laboratory of Molecular and Functional Imaging for Clinical Translation, The First Affiliated Hospital of Jinan University, Guangzhou, China; 12 Department of Hematology, Shenzhen People’s Hospital, Second Clinical Medical College of Jinan University, First Affiliated Hospital of Southern University of Science and Technology, Shenzhen, China; 13 Department of Hematology, The Seventh Affiliated Hospital, Sun Yat-sen University, Shenzhen, China; 14 Department of Hematology, The Third Xiangya hospital, Central South University, Changsha, China; 15 CAS Key Laboratory of Molecular Imaging, Institute of Automation, Chinese Academy of Sciences, Beijing, China; 16 National Engineering Research Center of Personalized Diagnostic and Therapeutic Technology, Xiangya Hospital, Changsha, China; 17 National Clinical Research Center for Geriatric Disorders (Xiangya Hospital), Central South University, Changsha, China; 18 Hunan Key Laboratory of Skin Cancer and Psoriasis, Xiangya Hospital, Central South University, Changsha, China; 19 Hunan Engineering Research Center of Skin Health and Disease, Xiangya Hospital, Central South University, Changsha, China; 20 Department of Dermatology, Xiangya Hospital, Central South University, Changsha, China

**Correction: BMC Cancer (2024) 24:458**

10.1186/s12885-024-12188-8

Following publication of the original article [1], the authors reported an error in the equal contribution statement. The statement should read as follows:  Xiaoping Yi, Huien Zhan, Jun Lyu and Juan Du contributed equally to this work. 

This correction article includes the corrected statement and the original article [1] has been corrected.
