# Supplementary material for: A chest CT-based nomogram for predicting survival in acute myeloid leukemia
Source: BMC Cancer. 2024 Apr 12;24:458. doi: 10.1186/s12885-024-12188-8 (PMC11010287; doi:10.1186/s12885-024-12188-8)
Supplement: Supplementary file 1 — Supplementary Material 1 [file 12885_2024_12188_MOESM1_ESM.docx]

**Supplementary file**

**Supplementary methods**

**Machine learning and deep learning**

Random survival forest (RSF) is a special form of random forest (RF) (1)and can be used for analysis of survival data, as previously described(2).

Deep learning model is based on the mainstream deep-learning framework PyTorch, comprising a feedforward neural network with three hidden layers(3). We used to represent the clinical feature predictor variable, with m = 16, k = 16, and l = 8, and we finally output the patient’s risk value. Batch normalization, a nonlinear activation layer, and a dropout layer were used between each hidden layer in order to increase the fitting ability of the model.

**Supplementary Tables**

**Supplementary Table 1**

Independent risk factors for CT-MSF model

| Variables | Multivariate analysis | | |
| --- | --- | --- | --- |
|  | HR | 95%CI | *P* value |
| Myosarcopenia |  |  |  |
| 1 |  |  |  |
| 2 | 1.965 | 1.393-2.774 | 0.0001 |
| 3 | 1.112 | 0.760-1.627 | 0.5851 |
| 4 | 1.197 | 0.814-1.762 | 0.3612 |
| ELN 2022 Risk (n, %) |  |  |  |
| Adverse |  |  |  |
| Intermediate | 0.8169 | 0.6161-1.0831 | 0.1600 |
| Favorable | 0.5874 | 0.3849-0.8964 | 0.0136 |
| BMT |  |  |  |
| Non- Transplantation |  |  |  |
| Transplantation | 0.7340 | 0.4973-1.0834 | 0.1196 |
| Age (median, range) | 1.0184 | 1.0084-1.0285 | 0.0003 |
| Speen-CTV | 0.9280 | 0.9061-0.9504 | <0.0001 |
| SF-CTV | 1.0459 | 1.0278-1.0642 | <0.0001 |
| RDW | 0.9904 | 0.9835-0.9974 | 0.0072 |
| HDL | 0.7001 | 0.4457-1.0997 | 0.1217 |
| TG | 1.1860 | 1.0306-1.3648 | 0.0173 |

**Supplementary Table 2**

Validation of CT-MSF model over ELN risk model

|  | NRI | IDI |
| --- | --- | --- |
| 1- year | 50.09% (26.03%-65.90%) | 7.91% (P＜0.001) |
| 2-year | 70.94% (38.38%-88.93%) | 17.49% (P＜0.001) |
| 3-year | 68.41% (42.14%-91.85%) | 17.86% (P＜0.001) |
| 5-year | 63.40% (35.99%-118.14%) | 16.77% (P＜0.001) |

NRI, net reclassification improvement.

IDI, integrated discrimination improvement.

**Supplementary Table 3**

Rates of BMT, CR and survival in CT-MSF high and low risk patients

| Characteristics | All samples (n=952) | CT-MSF risk Low (n=691) | CT-MSF risk High (n=261) | *P* value |
| --- | --- | --- | --- | --- |
| BMT |  |  |  | 0.0021 |
| Non- Transplantation | 755(79.31) | 507 (73.37) | 248 (95.02) |  |
| Transplantation | 197(20.69) | 184 (26.63) | 13 (4.98) |  |
| Status (n, %) |  |  |  | <0.001 |
| CR | 619 (65.02) | 504 (72.94) | 115 (44.06) |  |
| Non-CR | 333 (34.98) | 187 (27.06) | 146 (55.94) |  |
| Survival rate (%, CI) |  |  |  |  |
| 1-year |  | 75.54 (72.31-78.91) | 38.15 (32.50-44.79) | <0.001 |
| 2-year |  | 60.94 (57.06-65.09) | 17.85 (13.18-24.17) | <0.001 |
| 3-year |  | 53.80 (49.40-58.60) | 13.40 (8.86-20.40) | <0.001 |
| 5-year |  | 39.40 (32.20-48.30) | 8.96 (4.44-18.10) | <0.001 |

**Supplementary figures**


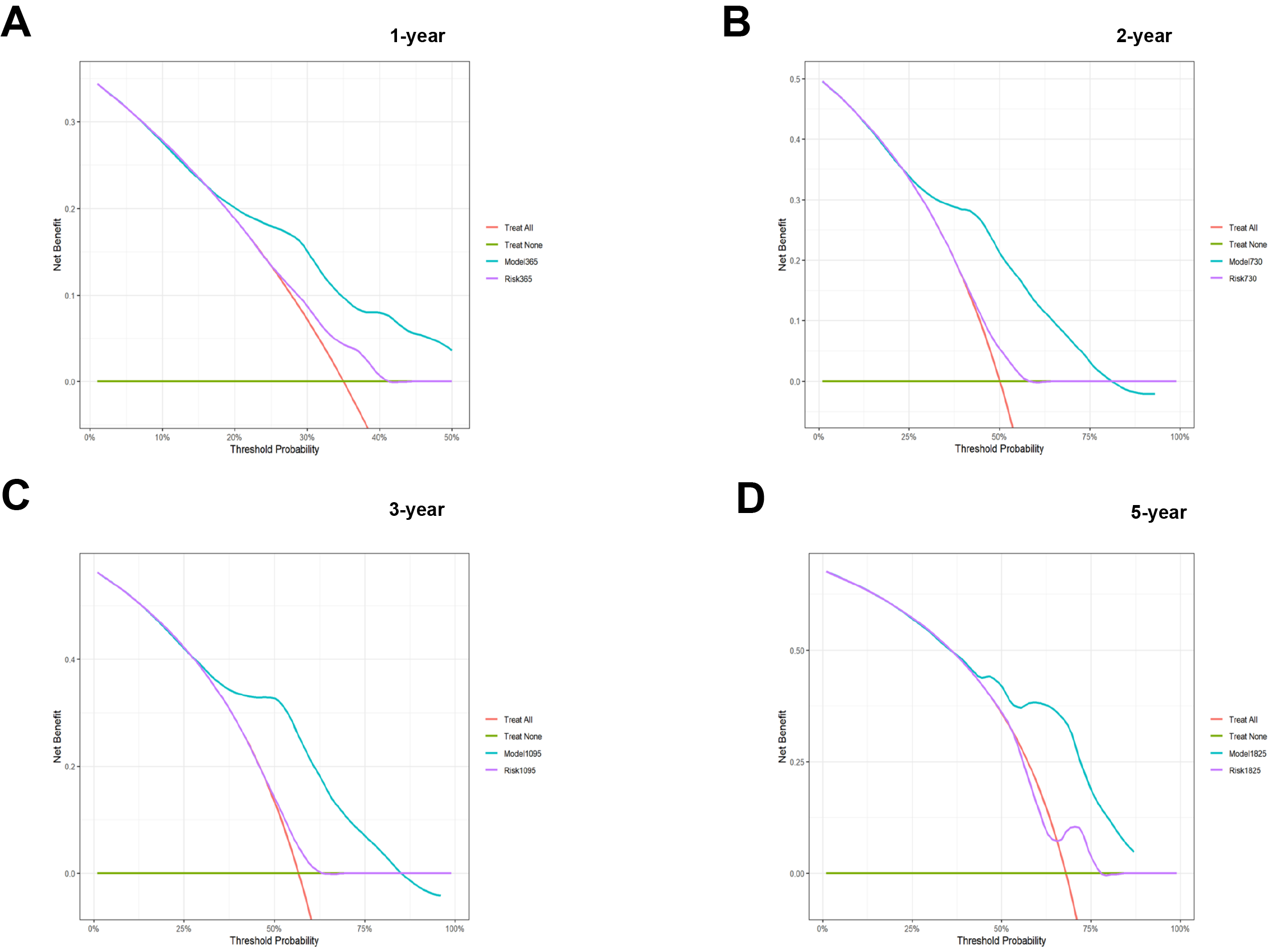


Figure S1. Decision curve analysis (DCA) of CT-MSF model (cyan) and ELN risk model (purple) at 1-(A), 2-(B), 3-(C) and 5- year (D). Analysis was done with the validation cohort composed of 476 patients.


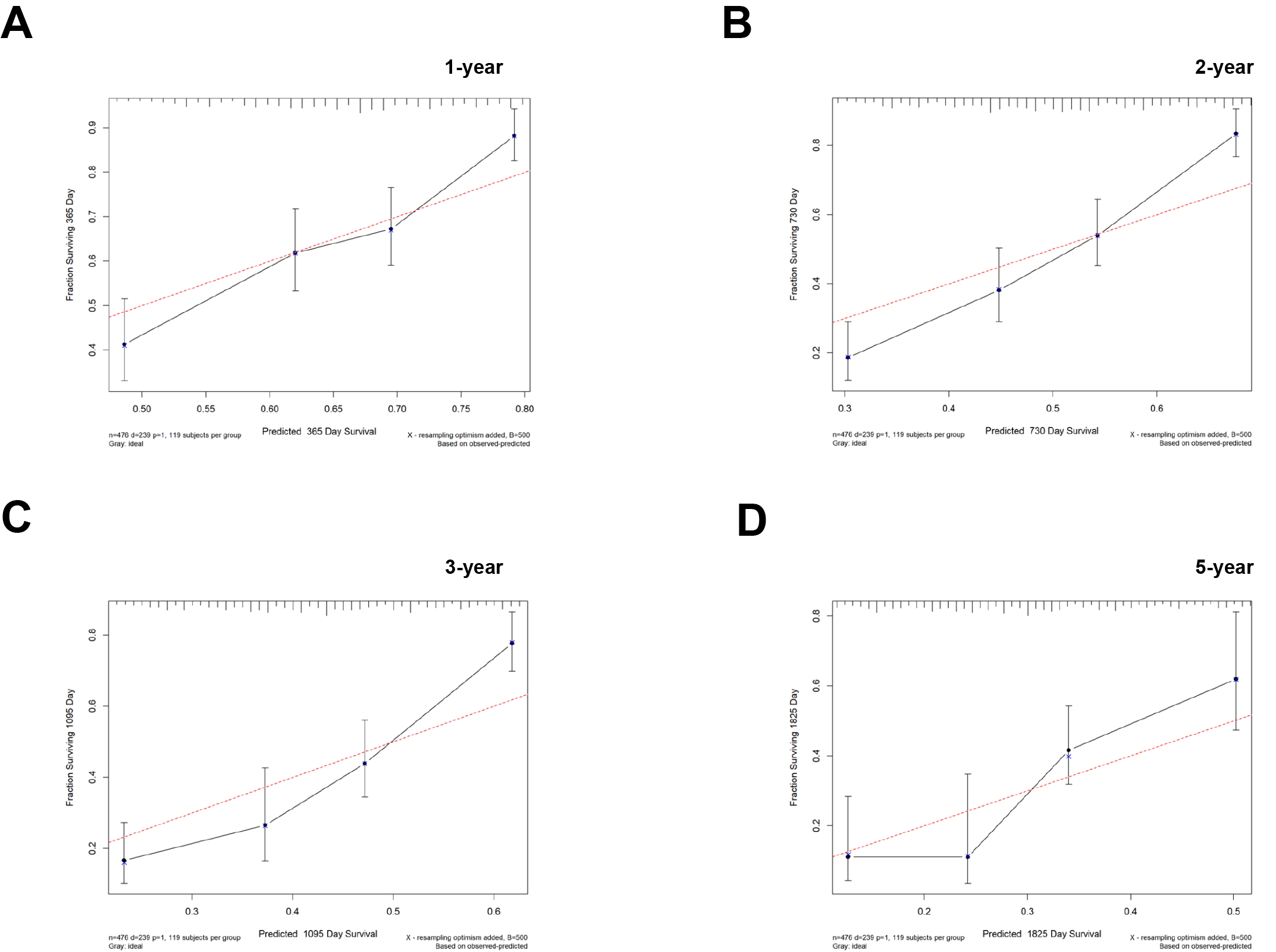


Figure S2. Calibration curve of CT-MSF model at 1(A), 2(B), 3(C) and 5- year(D). Analysis was done with the validation cohort. Calibration curves depict the calibration of the CT-MSF nomogram in terms of the agreement between the predicted probabilities and observed frequencies of the validation cohort.


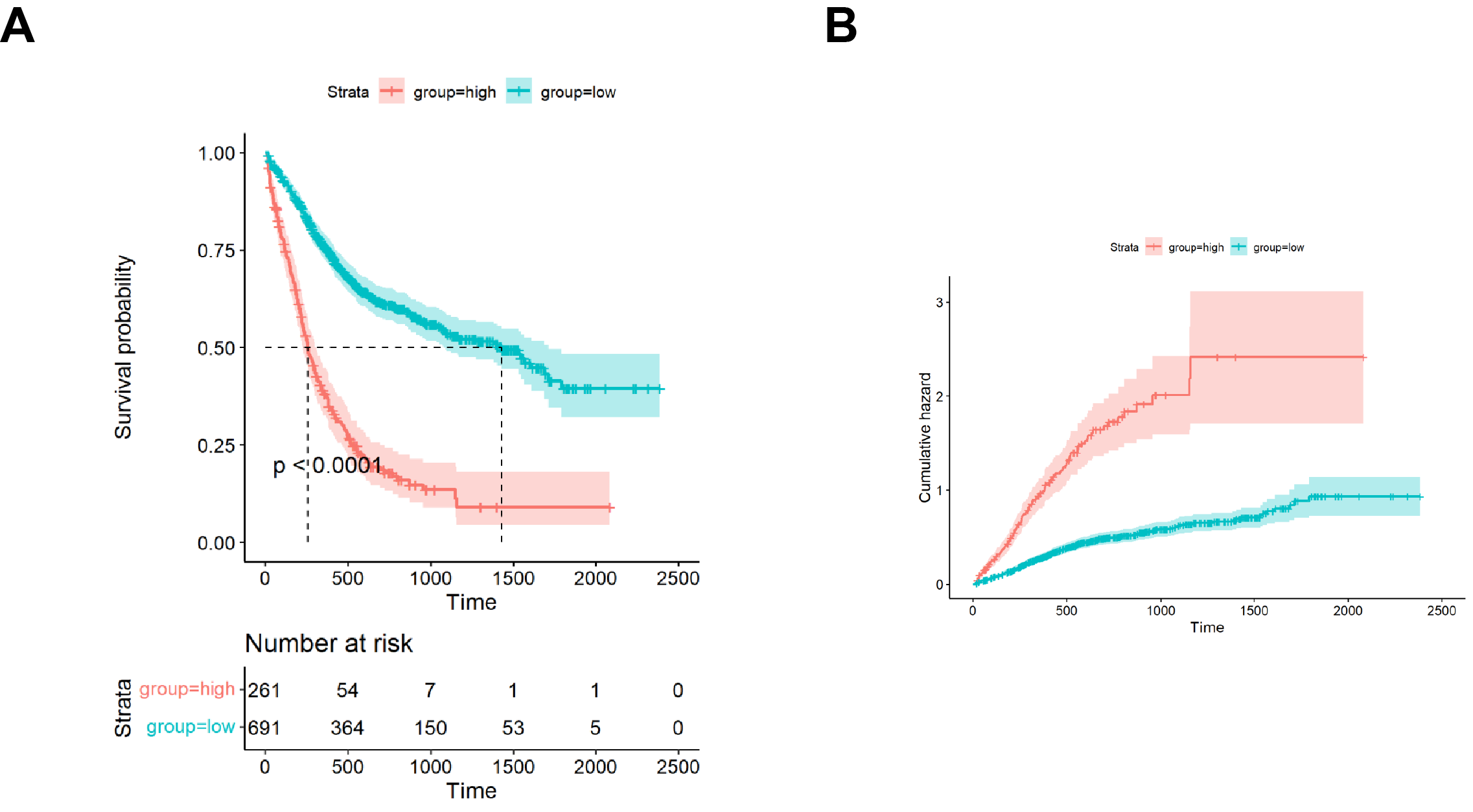


Figure S3. Prognosis prediction of the whole cohort comprising of 952 AML patients by CT-MSF model. (A) Overall survival of high-risk and low-risk patients stratified with CT-MSF model. (B) Cumulative hazard of high-risk and low-risk patients.


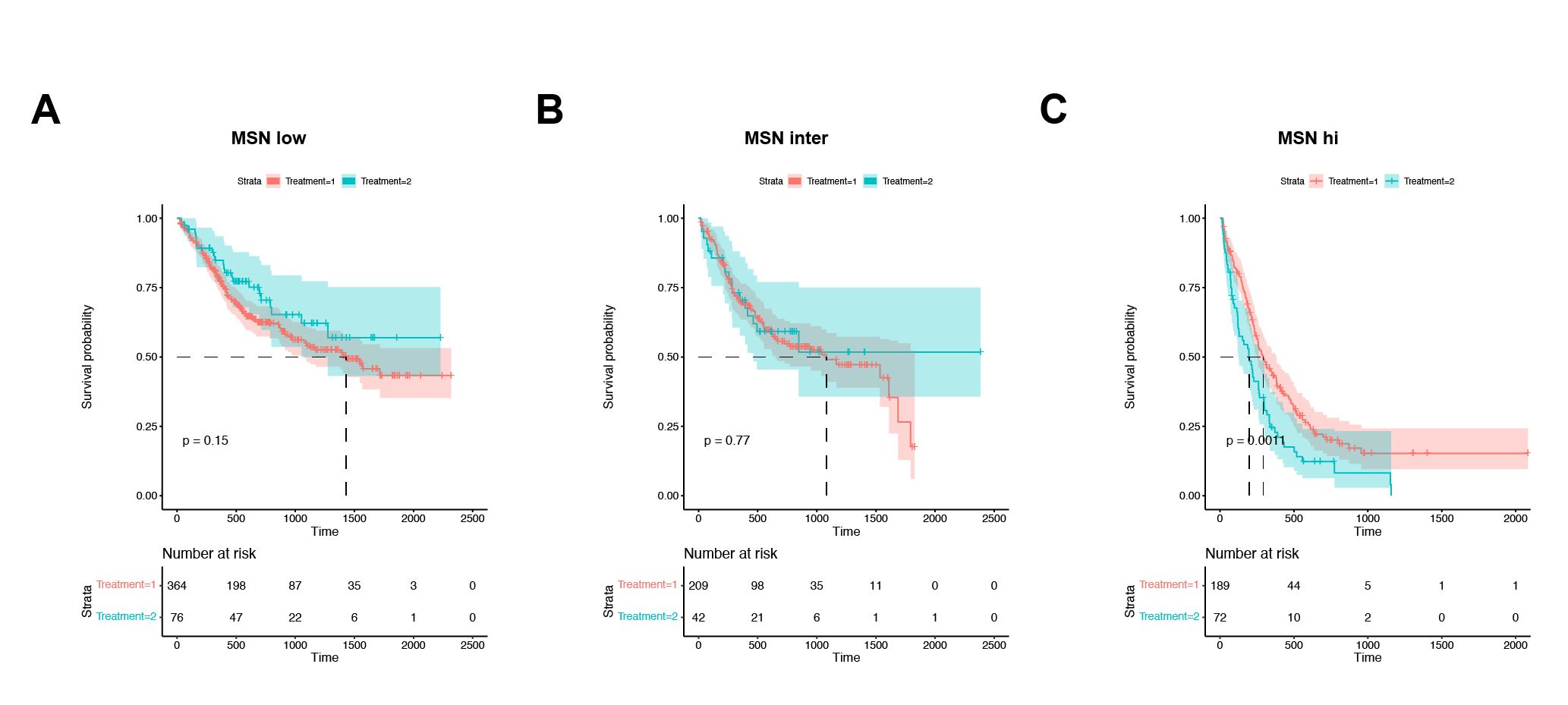


Figure S4. The MSN stratification system can help with clinical decisions. (A-C) Overall survival of patients with standard-dose treatment (treatment 1) or low-intensity treatment (treatment 2) in the MSN low-(A), intermediate-(B) and high-risk groups(C). MSN low risk, 440 patients. MSN intermediate risk, 251 patients. MSN high, 261 patients. A bilateral probability value of p < 0.05 was considered indicative of statistical significance.

**References**

1. Wu WT, Li YJ, Feng AZ, Li L, Huang T, Xu AD, Lyu J. Data mining in clinical big data: the frequently used databases, steps, and methodological models. Military Medical Research 2021, 8: 44.
2. Zhang LM, Huang T, Xu FS, Li SJ, Zheng S, Lyu J, Yin HY. Prediction of prognosis in elderly patients with sepsis based on machine learning (random survival forest). BMC Emergency Medicine 2022, 22: 26.
3. Yang R, Huang T, Wang ZC, Huang W, Feng AZ, Li L, Lyu J. Deep-Learning-Based Survival Prediction of Patients in Coronary Care Units. Computational and Mathematical Methods in Medicine 2021, https://doi.org/10.1155/2021/5745304.
